# Mesenchymal Stromal Cells and Exosomes: Progress and Challenges

**DOI:** 10.3389/fcell.2020.00665

**Published:** 2020-07-17

**Authors:** Matthew H. Forsberg, John A. Kink, Peiman Hematti, Christian M. Capitini

**Affiliations:** ^1^Department of Pediatrics, School of Medicine and Public Health, University of Wisconsin-Madison, Madison, WI, United States; ^2^Department of Medicine, School of Medicine and Public Health, University of Wisconsin-Madison, Madison, WI, United States; ^3^Carbone Cancer Center, University of Wisconsin-Madison, Madison, WI, United States

**Keywords:** MSCs, extracellular vesicles, exosomes, acute radiation syndrome, macrophages

## Abstract

Due to their robust immunomodulatory capabilities, mesenchymal stem/stromal cells (MSCs) have been used as a cellular therapy for a number of human diseases. Part of the mechanism of action of MSCs is the production of extracellular vesicles (EVs) that contain proteins, nucleic acids, and lipids that transmit signals to recipient cells that change their biologic behavior. This review briefly summarizes the development of MSCs as a treatment for human diseases as well as describes our present understanding of exosomes; how they exert their effects on target cells, and how they are differentiated from other EVs. The current treatment paradigm for acute radiation syndrome (ARS) is discussed, and how MSCs and MSC derived exosomes are emerging as treatment options for treating patients after radiation exposure. Other conditions such as graft-versus-host disease and cardiovascular disease/stroke are discussed as examples to highlight the immunomodulatory and regenerative capacity of MSC-exosomes. Finally, a consideration is given to how these cell-based therapies could possibly be deployed in the event of a catastrophic radiation exposure event.

## Infusion of MSCS for Treating Inflammatory Diseases

Mesenchymal stem/stromal cells (MSCs) are a potent immunomodulatory cell subset that are readily obtainable and easily expandable *in vitro*. MSCs can be obtained from many different tissues (bone marrow, adipose tissue, peripheral blood, umbilical cord blood), and are being studied for a number of conditions due to their ability to differentiate into various cell types, to migrate to various tissues, and to function as potent immunomodulators (Hass et al., 2011; Musiał-Wysocka et al., 2019). These cells are already approved in Europe for the treatment of complex perianal fistulas in adults with non-active/mildly active luminal Crohn’s disease (daradstrocel, Alofisel) and in Japan for steroid-refractory acute graft-versus-host-disease (GVHD) (TEMCELL). A Biologics License Application (BLA) has been submitted to the Food and Drug Administration (FDA) in the United States for steroid refractory acute GVHD in children (remestemcel-L, Ryoncil), with approval expected late 2020. Future BLAs may soon follow since MSCs have shown to be safe or exhibit clinical efficacy for the treatment of other highly inflammatory conditions such as chronic GVHD (Gao et al., 2016; Chen et al., 2019), ankylosing spondylitis (Wang P. et al., 2014), atopic dermatitis (Kim et al., 2017), bronchopulmonary dysplasia (Chang et al., 2014; Ahn et al., 2017), pulmonary emphysema (de Oliveira et al., 2017), non-ischemic cardiomyopathy (Chin et al., 2011; Butler et al., 2017), liver allograft rejection (Shi et al., 2017) and cirrhosis (Zhang et al., 2012; Suk et al., 2016; Liang et al., 2017), juvenile idiopathic arthritis (Swart et al., 2019), type 1 and type 2 diabetes (Jiang et al., 2011; Cai et al., 2016; Bhansali et al., 2017), rheumatoid arthritis (Park et al., 2018; Shadmanfar et al., 2018; Ghoryani et al., 2019), multiple sclerosis (Mohyeddin Bonab et al., 2007; Karussis et al., 2010; Bonab et al., 2012; Li J.F. et al., 2014; Harris et al., 2018; Riordan et al., 2018), systemic lupus erythematous (Wang D. et al., 2013, 2014), and osteoarthritis (Davatchi et al., 2011, 2016; Koh and Choi, 2012; Orozco et al., 2013; Wong et al., 2013; Vega et al., 2015; Lamo-Espinosa et al., 2016, 2018; Soler et al., 2016; Emadedin et al., 2018; Khalifeh Soltani et al., 2019; Matas et al., 2019).

## Infusion of MSCS for Tissue Repair and Regeneration

In part due to their immunomodulatory properties, MSCs have been observed to promote a regenerative environment that aids in the functional recovery of various damaged tissues (Bernardo et al., 2012). MSCs have proven to be safe or exhibited clinical efficacy in the field of regenerative medicine. Examples include improving neurologic function in amyotrophic lateral sclerosis (Petrou et al., 2016; Sykova et al., 2017; Berry et al., 2019), cerebral palsy (Wang X. et al., 2013; Huang et al., 2018), delayed encephalopathy after carbon monoxide poisoning (Wang H. et al., 2016), epilepsy (Hlebokazov et al., 2017), stroke (Bang et al., 2005; Lee et al., 2010), metachromatic leukodystrophy (Koc et al., 2002), and spinal cord injury (Vaquero et al., 2018); improved sexual function in erectile dysfunction (Al Demour et al., 2018); improved motor activity with frailty disorder (Tompkins et al., 2017), and multiple system atrophy (Lee et al., 2008, 2012; Singer et al., 2019); improved cardiovascular function in heart failure (Hare et al., 2012; Golpanian et al., 2015; Mathiasen et al., 2015; Bartolucci et al., 2017), and myocardial ischemia/angina (Hare et al., 2009; Friis et al., 2011; Haack-Sorensen et al., 2013; Karantalis et al., 2014; Kim et al., 2018), improved bone repair in hypophosphatasia (Taketani et al., 2015), lumbar disc degeneration (Orozco et al., 2011; Noriega et al., 2017), osteogenesis imperfecta (Gotherstrom et al., 2014), and osteonecrosis (Hernigou et al., 2018); improved healing from kidney injury (Tan et al., 2012; Saad et al., 2017); improved healing from liver injury related to acute on chronic hepatitis (Shi et al., 2012; Lin et al., 2017) and ischemic biliary lesions following liver transplantation (Zhang et al., 2017); improved hematopoietic recovery (Xiao et al., 2013; Zhang et al., 2013; Xiong et al., 2014); and accelerated wound healing (Falanga et al., 2007). From these indications, remestemcel-L is already in phase III trials for advanced heart failure and chronic low back pain.

## Potential Mechanisms of Action

Mesenchymal stem/stromal cells have been shown to suppress inflammation through direct cell-to-cell contact in inflamed tissues and through production of numerous anti-inflammatory molecules such as indoleamine 2,3 dioxygenase (IDO) (Su et al., 2014), nitric oxide (NO) (Su et al., 2014), prostaglandin E2 (PGE2) (Hsu et al., 2013), transforming growth factor (TGF)-β (de Araujo Farias et al., 2018), heme oxygenase 1 (HO1) (Chabannes et al., 2007), and hepatocyte growth factor (HGF) (Lee et al., 2018), among others. These molecules suppress the effect of immune cells such as macrophages (Nemeth et al., 2009; Eslani et al., 2018), monocytes (Cutler et al., 2010), dendritic cells (Jiang et al., 2005), B-cells (Corcione et al., 2006), NK cells (Sotiropoulou et al., 2006), and T-cells (Engela et al., 2013; Li M. et al., 2014). In addition to immuno-suppressive molecules, MSCs can influence target cell function through the secretion of large amounts of exosomes. MSC-derived exosomes have been investigated in preclinical models as a potential therapeutic for many of the same conditions that MSCs have shown efficacy in treating, such as wound healing (Fang et al., 2016; Samaeekia et al., 2018), angiogenesis (Teng et al., 2015; Huang et al., 2017), bronchopulmonary dysplasia (Braun et al., 2018), and various autoimmune disorders (Riazifar et al., 2019), but have also shown efficacy in facilitating skeletal muscle regeneration (Nakamura et al., 2015), neurogenesis (Reza-Zaldivar et al., 2019), recovery from stroke (Zhang and Chopp, 2016), and tendon repair (Chamberlain et al., 2019).

To date, no clinical trials infusing MSC-exosomes have been published although some studies have been completed (NCT03384433, NCT02138331) or are recruiting/about to open to accrual (Table 1).

**TABLE 1:** Clinical trials infusing MSC-derived exosomes.

Clinical trial number	Title	Sponsor
NCT03857841	A Safety Study of Intravenous Infusion of Bone Marrow Mesenchymal Stem Cell-derived Extracellular Vesicles (UNEX-42) in Preterm Neonates at High Risk for Bronchopulmonary Dysplasia	United Therapeutics
NCT04173650	A Safety Study of the Administration of MSC Extracellular Vesicles in the Treatment of Dystrophic Epidermolysis Bullosa Wounds	Aegle Therapeutics
NCT04276987	A Pilot Clinical Study on Aerosol Inhalation of the Exosomes Derived From Allogenic Adipose Mesenchymal Stem Cells in the Treatment of Severe Patients With Novel Coronavirus Pneumonia	Jiao Tong University School of Medicine Shanghai, China
NCT04213248	Effect of Umbilical Mesenchymal Stem Cells Derived Exosomes on Dry Eye in Patients With Chronic Graft Versus Host Diseases	Sun Yat-sen University Guangdong, China
NCT04313647	A Tolerance Clinical Study On Aerosol Inhalation of Mesenchymal Stem Cells Exosomes In Healthy Volunteers	Jiao Tong University School of Medicine Shanghai, China
NCT03437759	Mesenchymal Stem Cells Derived Exosomes Promote Healing of Large and Refractory Macular Holes	Tianjin Medical University Hospital Tianjin, China
NCT04270006	Effect of Adipose Derived Stem Cells Exosomes as an Adjunctive Therapy to Scaling and Root Planning in the Treatment of Periodontitis: A Human Clinical Trial	Beni-Suef University Beni-Suef, Egypt

*According to clinicaltrials.gov as of 14 April 2020.*

This review focuses on recent pre-clinical work on the potential therapeutic uses of MSCs and MSC-exosomes to polarize or “educate” immune cells into anti-inflammatory cells, with treatment of acute radiation syndrome (ARS) and GVHD as models for the systemic effects of the anti-inflammatory properties of MSC-exosomes. The organ-specific regenerative effects of MSC-exosomes are also explored, using cardiovascular disease and stroke as examples. ARS is also used as an example of how MSC derived exosomes could be developed as a cell-based therapeutic, with consideration given to the potential challenges and drawbacks of such an approach. For a thorough review on how MSC exosomes are being used for the treatment of other conditions, a recently published review is highly recommended (Joo et al., 2020).

## Exosomes: Formation, Characteristics, and Cargo

Extracellular vesicles (EVs) are lipid bilayer particles that are released from cells. This diverse family of particles includes microvesicles (MVs), apoptotic bodies, and exosomes. EVs are composed of membrane-bound particles that are classified according to size with exosomes generally defined as 30–150 nm in diameter (Helwa et al., 2017; Bebelman et al., 2018). Exosomes are generally considered to be produced through the inward budding of late stage endosomes, forming multi-vesicular bodies (MVBs) which then release these “buds” (exosomes) upon fusion with the plasma membrane (Hessvik and Llorente, 2018). Due to the fact that exosomes are differentiated from EVs based on their relative size, it can be difficult to separate exosomes from smaller EVs. In fact, the formation of a smaller subset of EVs in the size range of what is considered to be exosomes has been observed through the direct budding of the plasma membrane (Casado et al., 2017). Cholesterol, sphingomyelin, ceramide, and various lipid molecules are found in large quantities on the exosomal membrane (Mashouri et al., 2019). Once the exosomes are released into the intercellular space, they can be taken up by recipient cells by endocytosis, receptor–ligand binding, or through direct binding (Kahroba et al., 2019).

**FIGURE 1:**
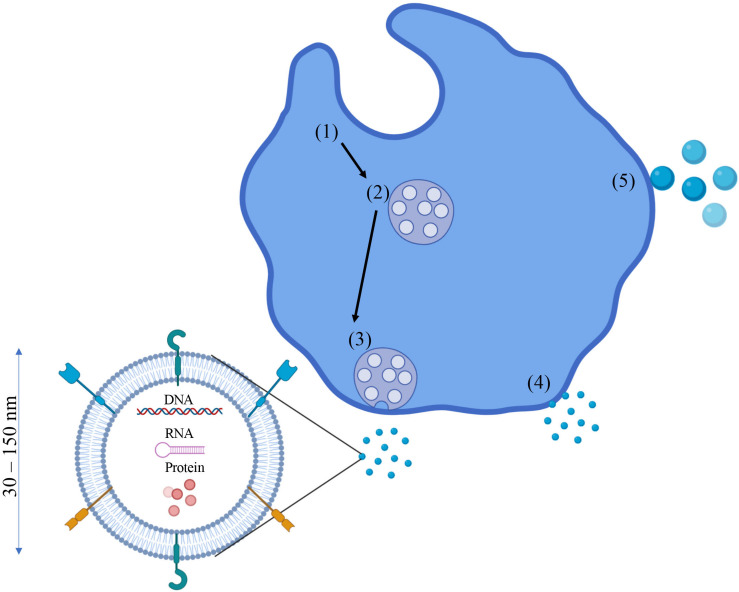
Exosomes: Formation, cargo, and characteristics. Exosome formation typically begins as endosomes (1) begin to bud inward and form multi-vesicular bodies (MVBs) (2). These MVBs then fuse with the plasma membrane (3) and release the exosomes into the intracellular space. However, the plasma membrane can also bleb off small EVs 30–150 nm in diameter which also fall in the same size classification as exosomes, so differentiating the two can be difficult (4). If these plasma membrane blebs are > 150 nm in diameter, they are classified as microvesicles or microparticles (5). Exosomes can contain a number of different molecules as cargo such as proteins/cytokines (free floating and membrane bound), DNA, RNA, and other nucleic acids.

Exosomes exert their effects by releasing their contents into the cytosol of recipient cells. Exosome cargo can consist of a number of different molecules such as nucleic acids (DNA, RNA, mRNA miRNA), pro-inflammatory and anti-inflammatory cytokines, enzymes, and various other proteins (Mathivanan et al., 2010; D’Asti et al., 2012). Cytokines can be found not only encapsulated in the exosome, but also imbedded in the exosomal membrane itself (Fitzgerald et al., 2018). The authors of this study hypothesized that exosomes can deliver smaller amounts of cytokines directly to the intended target cell, a more efficient delivery mechanism compared to the traditional cytokine “dump” into the intercellular space (which could be taken up by any cell with a corresponding receptor). Other proteins found in the exosomal membrane such as various heat-shock and signaling proteins have shown to perform immunomodulatory functions as well (Urbanelli et al., 2013; Reddy et al., 2018). A visual representation of exosomes, their formation, and their cargo can be seen in Figure 1.

## Potential Benefits and Challenges of Using MSC-Exosomes

Utilizing MSC-exosomes as a therapy has a number of advantages compared to using MSCs themselves. One advantage is that viability is not a concern with exosomes, as they are not cells. This makes exosomes potentially much easier to use post thaw. Indeed, there is preliminary evidence that the thawing process may alter exosomal membranes so that they are absorbed more easily by target cells, although more research is needed to confirm this finding (Cheng et al., 2019). In one report, familial patients who received autologous adipose tissue-derived MSCs all had incidences of pulmonary embolisms related to the infusion (Jung et al., 2013). As MSC-exosomes are not a cellular product, there should be no risk to potential patients of developing pulmonary embolisms. Exosomes can also cross the blood–brain barrier, while MSCs cannot, making MSC-exosomes an attractive potential therapy option for various degenerative brain disorders (Chen et al., 2016). Just as with any novel therapy, there are challenges that need to be addressed before MSC-exosomes can be used in the clinic. First and foremost there needs to be a method of selecting suitable donors for the production of the MSCs that are used to produce exosomes, as well as the development of an exosome isolation protocol that meets good manufacturing practice (GMP) standards. Additionally, a consistent release criterion (size, surface marker expression, cargo, etc.) needs to be established for the exosomes themselves before they can be infused into potential patients. Depending on the condition being treated, markers need to be identified that distinguish functional from non-functional exosomes. Also, the optimal dose of MSC-exosomes for humans is unknown, which would need to be determined for each condition being treated. Furthermore, the best route of administration (local versus systemic) is unclear, as is the length of time MSC-exosomes remain in the patient before they are cleared by phagocytic cells. These challenges must be overcome and standards must be defined before patients can be tested for the immunomodulatory and regenerative capabilities of MSC-exosomes.

## MSC Derived Exosomes for the Treatment of ARS

Acute radiation syndrome is caused by a high dose of ionizing radiation (>1 GY) over a short period of time (Lopez and Martin, 2011). The most severe side effects of ARS occur due to damage in highly proliferative cells found in the skin, the gastrointestinal tract, and the bone marrow (Heslet et al., 2012). Loss of bone marrow progenitor cells places patients at high risk for infections, as they can no longer produce leukocytes (Dainiak, 2018). The current standard of care for ARS involves the use of supportive care measures such as prophylactic antibiotics, blood and platelet transfusions, and the growth factors granulocyte colony-stimulating factor (G-CSF) and granulocyte-macrophage colony-stimulating factor (GM-CSF) (Waselenko et al., 2004; Gourmelon et al., 2010) which are FDA-approved to treat ARS. These interventions can keep patients alive and provide valuable time to patients who are waiting for an allogeneic hematopoietic stem cell transplant (HSCT) (Weisdorf et al., 2006). However, this process can take several weeks, during which time the patient may die from the initial exposure event. Even if the patient successfully receives a HSCT, this procedure comes with its own set of risks such as engraftment failure and GVHD (Ghimire et al., 2017; Ozdemir and Civriz Bozdag, 2018).

**FIGURE 2:**
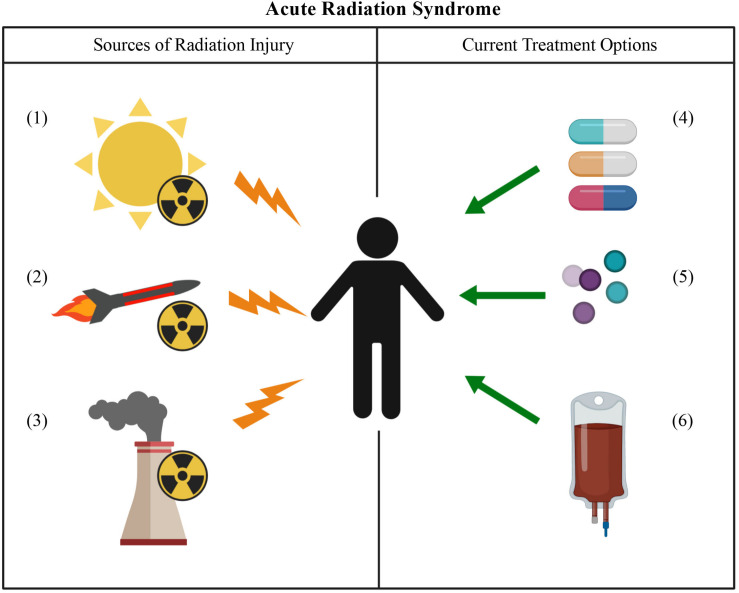
Acute radiation syndrome: sources of injury and current treatment options. Sources of radiation injury can include solar flares (1), nuclear weapons (2), or an accident at a nuclear power plant (3). The current treatment options for ARS involve the use of antibiotics (4) and the growth factors G-CSF and GM-CSF (5). In the more severe cases of ARS, an HSCT may need to be performed (6).

Most cases of ARS are seen in nuclear power plant employees upon accidental exposure resulting from incidents occurring at the plants such as those seen at Chernobyl and Fukushima (Mettler et al., 2007; Cerezo and Macia, 2012). Increasing usage of medical isotopes like iodine-131 to treat cancer has resulted in patients needing to cryopreserve their own (autologous) hematopoietic stem cells prior to treatment so that the bone marrow can be rescued from the cancer treatment. However, due to the current proliferation of nuclear technology worldwide, a mass exposure event from a terrorist attack using an improvised nuclear device or from a nuclear warhead deployed as an act-of-war is also a possibility. ARS could also impact future astronauts as both government agencies like the National Aeronautics and Space Administration (NASA) and private companies push for human voyages back to the moon as well as to Mars. An event such as a solar flare could expose astronauts to a high dose of cosmic radiation (Chancellor et al., 2014). Possible sources of radiation exposure, as well as current treatment options are summarized in Figure 2. Due to the current standard of care for ARS as well as the increased risk of exposure events due to accidents at power plants, cancer treatments, political instability, or through the colonization of the inner solar system; a priority has been placed on the development of “off-the-shelf” cell-based therapies that seek to mitigate or even reverse the deleterious effects of ARS.

A number of preclinical studies have shown that MSCs can be used to reverse radiation damage seen in a variety of tissues, including the bone marrow (Fukumoto, 2016). The majority of preclinical work has been done utilizing bone marrow-derived MSCs for treatment of ARS (Lange et al., 2011; Yang et al., 2012). However, adipose tissue derived MSCs have also shown to prolong survival in irradiated mice, as well as enhance the reconstitution of hematopoietic cells (Cousin et al., 2003). Intramuscularly injected human placenta-derived MSCs have also shown the ability to enhance hematopoietic regeneration, reverse severe weight loss, and increase survival in lethally irradiated mice (Gaberman et al., 2013; Pinzur et al., 2018). Interestingly, in a study that exposed C57/BL6 mice to lethal irradiation followed by subsequent treatment with bone marrow-derived syngeneic MSCs within 24 h after exposure, the infused MSCs were cleared from the recipient mice within 3 days (Yang et al., 2012). This finding suggests that the protective effect seen in these mice was not due to the infused MSCs themselves, but rather through endogenous cells that were “educated” by the MSCs. One way in which these target cells could have been educated was by paracrine factors such as EVs produced from MSCs. Indeed, sublethally irradiated C57/BL6 lineage negative bone marrow cells (isolated 7 days after irradiation) showed an increased capacity to engraft in syngeneic recipient mice after being cultured with murine or human MSC-EVs (Wen et al., 2016).

Presently, only one clinical trial using allogeneic ex vivo expanded placental MSCs (PLX-R18, Pluristem Ltd.) is available for ARS, but is not yet recruiting (NCT03797040). However as stated above, it is possible that the benefits of MSCs in ARS may not be from MSCs themselves. Rather, the MSCs may be educating other immune cell subsets such as macrophages to mediate their radioprotective effects (Davies et al., 2017). Human MSC-educated macrophages, or “MEMs,” are a high interleukin (IL)-6 and IL-10 producing macrophage subset (Kim and Hematti, 2009) that are more effective than human MSCs alone in treating ARS in an immunocompromised xenogeneic model (Bouchlaka et al., 2017). In this study, MEMs expressed higher levels of inhibitory molecules such as PD-L1 and PD-L2, as well as molecules CD73 and arginase-1, when compared to untreated macrophages. Furthermore, MEMs were found to secrete higher levels of IL-6, while exhibiting a greater capacity to inhibit the proliferation of T-cells while promoting fibroblast proliferation as compared to untreated macrophages. IL-6 is typically considered to be a pro-inflammatory cytokine (Tanaka et al., 2014), although it has also been found in some instances to have anti-inflammatory effects as well as playing a role in tissue regeneration (Scheller et al., 2011; Galun and Rose-John, 2013). In fact, the presence of IL-6 has been associated with reduced inflammation caused by radiation induced injury (Bell et al., 2019). This increased secretion of IL-6 was enhanced when MEMs were “primed” with the TLR-4 ligand lipopolysaccharide (LPS) (Bouchlaka et al., 2017). The role of LPS in promoting the radio-protective effect of MEMs was investigated in a recent study, where LPS induced generation of MSC derived exosomes in a dose-dependent manner (Kink et al., 2019). In this study, human bone marrow-derived MSCs were primed with LPS for 24 h, after which time exosomes were harvested (Kink et al., 2019). The LPS-MSC exosomes (and unprimed MSC exosomes) were then cultured with human macrophages for 3 days, followed by infusion into immunocompromised mice with ARS. Results from this study show that the LPS-primed MSC exosome educated macrophages (LPS EEMs) significantly prolonged survival from ARS, enhanced hematopoietic recovery (by complete blood count and histologic analysis of bone marrow and spleen), and increased phagocytic capacity compared to unprimed MSC exosome educated macrophages (EEMs). The mechanism behind this protective effect is currently unknown, although there are a few intriguing avenues to investigate. The protection may be due to the effects of the increased levels of IL-6 or enhanced expression of PD-L1 found on the surface of the LPS EEMs. This immunomodulatory environment could possibly facilitate the reconstitution of the host mouse bone marrow. In order to investigate these possibilities, researchers could perform a similar study to those mentioned above, except for the inclusion of the administration of IL-6 and/or PD-L1 blocking monoclonal antibodies and observe how the radio-protective effect of the LPS-EEMs is altered. Furthermore, the use of LPS causes some concerns in a clinical setting, as LPS is toxic to humans (Stewart et al., 2006). Therefore, it would be prudent to perform similar experiments using synthetic LPS analogs in place of LPS.

## MSC Derived Exosomes for the Treatment of GVHD and COVID-19

Due to their immunomodulatory capabilities, MSC-exosomes are an intriguing potential therapy for GVHD. Similar to ARS, GVHD is characterized by damaging proinflammatory responses that affect multiple organs. Immunosuppression with corticosteroids remains the most common treatment for both acute and chronic GVHD, but with sustained response rates around 40–50%, more effective approaches are needed (Garnett et al., 2013). Recent preclinical studies have investigated the use of human MSC-exosomes in mice with acute GVHD. Both umbilical cord derived MSC-exosomes and BM derived MSC exosomes prolonged survival of mice with GVHD compared to controls (Wang L. et al., 2016; Fujii et al., 2018). Furthermore, these studies showed a lower number of T cells, impaired T cell proliferation, and lower levels of proinflammatory cytokines IL-2, TNF-α, and IFN-γ in MSC-exosome treated mice (Wang L. et al., 2016; Fujii et al., 2018). Human BM derived MSC-exosomes have also shown efficacy in mouse models of chronic GVHD. In this study, treated mice showed improved survival, diminished clinical scores, reduced fibrosis in the skin, lung and liver, inhibition of Th17 cells, and induction of IL-10 expressing regulatory cells (Lai et al., 2018). Interestingly, BM derived MSC-exosomes have been given to a human patient with severe therapy-resistant GVHD as an individualized compassionate use treatment (Kordelas et al., 2014). The patient responded well to the therapy, as patient PBMCs showed decreased production of IL-1β, TNF-α, and IFN-γ after the third infusion (Kordelas et al., 2014). Additionally, clinical GVHD symptoms improved significantly, which allowed for a reduced dosage of steroids (Kordelas et al., 2014). The patient remained stable for months before eventually dying 7 months later due to pneumonia (Kordelas et al., 2014). A more recent finding, although not GVHD related, also shows the impact that MSC-exosomes have on reducing inflammatory responses; 24 severe COVID-19 patients were given a dose of ExoFlo^TM^ (a BM derived MSC-exosome product) at a single hospital center (Sengupta et al., 2020). Of these 24 patients, 17 fully recovered, three died, and three remain in intensive care at the time of publication (Sengupta et al., 2020). There were no adverse reactions to ExoFlow^TM^ seen in any of the patients (Sengupta et al., 2020). Patients showed a significant decrease in neutrophil count, an increase in T lymphocyte count, reversal of hypoxia, and downregulation of cytokine storm (Sengupta et al., 2020). More clinical work is needed to determine if MSC-exosomes constitute an effective therapy for inflammatory diseases, but initial results are promising.

## MSC Derived Exosomes for the Treatment of Cardiovascular Disease and Stroke

Cardiovascular diseases are the leading cause of morbidity and mortality worldwide. One major cause/result of these conditions is the death of cardiomyocytes and the subsequent loss of tissue remodeling capabilities (Olivetti et al., 1996). Cell-based therapies, including MSCs, have been investigated as a potential therapeutic option to help replace the lost cardiomyocytes, and improve heart function (Golpanian et al., 2016; Majka et al., 2017). MSC-exosomes have also shown promise in promoting cardioprotection in a mouse model of myocardial ischemia/reperfusion (I/R) injury, evidenced by reduced infarct size in mice treated with purified MSC-exosomes (Lai et al., 2010). Similarly, in a rat model of myocardial ischemic injury, human umbilical cord MSC-exosomes were found to reduce cardiac cell fibrosis, suppress apoptosis, and promote proliferation (Zhao et al., 2015). In a different study, researchers found that this protective effect of MSC-exosomes was in part due to their ability to deliver miRNAs, specifically miR-19a (Yu et al., 2015). Enhanced myocardial viability has also been observed in an I/R injury mouse model, resulting from increased ATP levels and decreased oxidative stress seen in the heart tissue of mice treated with MSC-exosomes (Arslan et al., 2013). Like cardiovascular disease, stroke is one of the leading causes of death and disability. MSC therapy has shown preclinical and clinical success in promoting recovery from stroke (Zhang and Chopp, 2016), and MSC-exosomes have shown promise in various preclinical models of stroke recovery. In rat models of traumatic brain injury (TBI), administration of MSC-exosomes has been shown to enhance neurogenesis and angiogenesis, and improve spatial learning and sensorimotor functional recovery (Xin et al., 2013a; Zhang et al., 2015). One potential mechanism of the enhanced recovery from TBI is the delivery of the miRNA miR-133b, as MSC-exosomes with elevated miR-133b led to improved axonal remodeling and neurological function when compared to standard MSC-exosomes (Xin et al., 2013b). MSC-exosomes promote tissue repair/remodeling in a number of preclinical disease models in addition to cardiovascular disease/stroke, and provide an exciting potential therapy for patients who suffer from these debilitating conditions.

## Implementation of Cell-Based Therapies for ARS

Considerations on how MSCs/MSC-exosomes can be best utilized need to be taken into account on a condition by condition basis. Here, ARS is used as an example to explore what hurdles need to be cleared before MSCs/MSC-exosomes can be used as a treatment for this condition. Cell-based treatment strategies for ARS need to be safe and efficacious in the event of a potentially lethal radiation exposure. To achieve this goal, a number of logistical challenges need to be taken into account. In order to outline the nature of these challenges, and develop strategies to overcome them, the National Institute of Allergy and Infectious Diseases (NIAID) co-sponsored an international workshop in July 2015 in Paris, France, with the Institut de Radioprotection et de Sûreté Nucléaire. A report on this workshop was published in Radiation Research (DiCarlo et al., 2017). In this report, the authors summarize the numerous regulatory hurdles that need to be cleared in order for these cell-based therapies to be approved for human use. Of particular interest was the potential of MSC produced paracrine factors, particularly exosomes, to treat the effects of radiation exposure (Tran et al., 2013). However, the report stated that both cell-based therapies as well as exosomes alone could be used in a mass exposure event (DiCarlo et al., 2017). Patients who were exposed to higher levels of radiation could receive MSCs or MSC exosome educated cells, while the larger number of patients exposed to lower radiation levels (who still have surviving hematopoietic stem cells) could receive MSC exosomes alone to help boost the capacity of their own bone marrow to replenish itself. Importantly, in both of these scenarios, the patient would be receiving an “off-the-shelf” thawed product, so the development of a consistent and effective post-thaw procedure prior to infusion is of upmost importance. Cell-based therapies would likely be stored in a cryopreserved state at a few centralized locations. Therefore, therapeutic cells would need to show efficacy up to 24 or 48 h after the initial radiation exposure in order to account for the time it would take for the product to reach the patient.

## Conclusion

Mesenchymal stem/stromal cell-exosomes are an emerging treatment for a variety of inflammatory and degenerative conditions, and are beginning to be translated from preclinical models into early phase clinical trials. For ARS, not only will therapies like MSCs, MSC-exosomes, and MSC-educated/MSC exosome-educated macrophages need to be tested for safety and efficacy, but they will also need to retain their function after cryopreservation/thawing so that supplies could be added to a National Stockpile or be transported on a space shuttle. Testing these cell-based options will either require clinical trials in patients with cancer receiving molecularly targeted radioactive therapies that show hematopoietic toxicity requiring growth factors, transfusions or HSCT, or become approved through mechanisms that bypass testing in patients like the FDA two-animal rule (Singh and Olabisi, 2017). Likewise, biomanufacturing standards need to be defined and standardized release criteria need to be developed for MSC-exosomes as they are used to treat other inflammatory conditions such as GVHD or COVID-19, as well as degenerative conditions such as cardiovascular disease and stroke. Depending on the indication, different potency assays may need to be used to verify anti-inflammatory versus tissue regenerative properties of MSC-exosomes. For these milestones to be met, increasing support from government agencies like NIAID, Department of Defense, NASA, and FDA will be needed to insure successful biomanufacturing of MSC-exosomes.

## Author Contributions

MF drafted the manuscript. JK, PH, and CC revised the manuscript. All authors approved the final version of the manuscript.

## Conflict of Interest

PH and CC are inventors on patents related to this publication. CC reports honorarium from Nektar Therapeutics, who had no input in the study design, analysis, manuscript preparation, or decision to submit for publication. The remaining authors declare that the research was conducted in the absence of any commercial or financial relationships that could be construed as a potential conflict of interest.
